# IoT-Based Strawberry Disease Prediction System for Smart Farming

**DOI:** 10.3390/s18114051

**Published:** 2018-11-20

**Authors:** Sehan Kim, Meonghun Lee, Changsun Shin

**Affiliations:** 1 loT Research Division, Electronics and Telecommunications Research Institute, Daejeon 34129, Korea; shkim72@etri.re.kr; 2 Department of Agricultural Engineering, National Institute of Agricultural Sciences, Jeollabuk-do 55365, Korea; 3 Department of Information and Communications Engineering, Sunchon National University, Jeollanam-do 57922, Korea

**Keywords:** smart farming, prediction, infection forecast model, IoT, oneM2M, LoRa

## Abstract

Crop diseases cannot be accurately predicted by merely analyzing individual disease causes. Only through construction of a comprehensive analysis system can users be provided with predictions of highly probable diseases. In this study, cloud-based technology capable of handling the collection, analysis, and prediction of agricultural environment information in one common platform was developed. The proposed Farm as a Service (FaaS) integrated system supports high-level application services by operating and monitoring farms as well as managing associated devices, data, and models. This system registers, connects, and manages Internet of Things (IoT) devices and analyzes environmental and growth information. In addition, the IoT-Hub network model was constructed in this study. This model supports efficient data transfer for each IoT device as well as communication for non-standard products, and exhibits high communication reliability even in poor communication environments. Thus, IoT-Hub ensures the stability of technology specialized for agricultural environments. The integrated agriculture-specialized FaaS system implements specific systems at different levels. The proposed system was verified through design and analysis of a strawberry infection prediction system, which was compared with other infection models.

## 1. Introduction

Smart farms are farms that can remotely and automatically control the growth environments of crops and livestock, by combining information and communications technology (ICT) with greenhouses, livestock barns, and orchards [1,2]. Such farms measure and analyze the temperature, humidity, and sunshine levels of farm facilities using technology such as the Internet of Things (IoT) and implement remote control through mobile devices [3,4,5]. To date, cutting-edge information technology (IT) applications such as Big Data, cloud computing, and robotics have been utilized for agriculture, and ICT integration, research, and development have been performed [6,7,8,9,10,11].

Smart farms are attracting global attention as a solution to the increasing demand for food due to population growth; labor force shortage owing to fewer and aging farmers; and ongoing problems in agriculture, such as limited distribution and expansion of cutting-edge agricultural technology [12,13,14]. During the last century, increases in temperature and rainfall due to sudden climate change posed significant problems for agriculture [15]. In particular, decreasing production owing to disease requires attention because this problem directly affects the income of farm households [16].

Many efforts toward disease prevention have recently been made [17,18]. Notably, the physiologies of the major pathogens affecting different crops have been determined, and the temperature dependence of disease occurrence has been identified using advanced technology and data analysis [19,20]. In addition, modeling capable of predicting disease occurrence has been performed through real-time collection of weather data using unmanned observation planes, as well as analysis of long-term recorded data [21,22].

In this study, the cloud-based Farm as a Service (FaaS) system is proposed, which is capable of handling the collection, analysis, and prediction of agricultural environment information on one common platform. The proposed FaaS is a common platform based on the oneM2M standards and supports openAPI (where API stands for “application programming interface”). Thus, various application services can be developed and collection, control, operation, and management of agricultural data are facilitated [23,24,25]. FaaS virtualizes the resource information of smart farms in various forms based on the Platform as a Service (PaaS) approach and provides environments for service operation and development [26,27]. The smart farm service established by FaaS utilizes cloud-based technology that shares IT infrastructure resources, such as servers, storage, middleware, and networks, through a network. FaaS also virtualizes smart farm devices, such as IoT devices and actuators, for operation. Existing heterogeneous smart farm systems installed and operated by farm households, as well as legacy systems installed and operated individually or separately by different suppliers, can be integrated in FaaS through cloud-based technology, and the farm management function can be used in the form of a cloud service. The proposed smart farm service can provide crop growth information monitoring services according to the requirements of farm households, as well as disease prediction services, through application of cloud computing, IoT, Big Data, and mobile technology to greenhouses, orchards, and livestock barns [28,29].

As a case study, Seolhyang, a South Korean strawberry variety, was cultivated. A model predicting the infection risk of *Botrytis cinerea*, a major disease, was then developed to evaluate the performance of the proposed system [30,31]. *Botrytis cinerea* generally causes damage to the fruit, calyxes, fruit stalks, leaves, and petioles of strawberries. In particular, it causes considerable damage to the fruit by invading young fruit and causing light or dark browning. In addition, this disease causes decomposition and gray pathogens under humid conditions.

The remainder of this study is organized as follows. In Section 2, the IoT-Hub design is presented. The FaaS system is classified into three layers: the network layer, FaaS layer, and application layer; the IoT-Hub network model supports the oneM2M platform, which is the low-level layer. The Long Range (LoRa) communication system is also discussed. In Section 3, the FaaS configuration and functions are described. FaaS is the middle-level layer. In Section 4, the overall system implementation and performance evaluation are discussed, and an infection prediction model specialized for strawberry diseases is developed. Thus, a technological application that utilizes the disease prediction system is presented. Finally, analysis and utilization of the developed system are discussed in the conclusion section.

## 2. IoT-Hub for FaaS

The FaaS device management service aims to provide interoperability among numerous IoT devices, even those based on platforms developed according to different standards or technology. In other words, it provides a common platform structure that is application-independent and that can support all services in the corresponding agricultural field.

### 2.1. IoT-Hub Network Model

**Figure 1:**
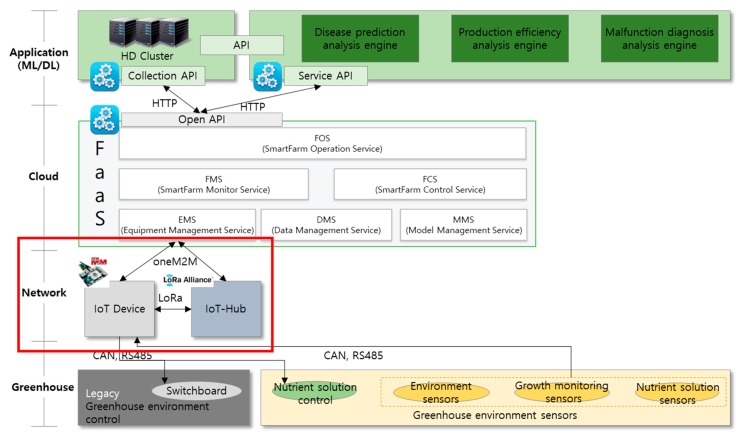
Network model using Internet of Things (IoT)-Hub. FaaS: Farm as a Service; api: application programming interface; LoRa: Long Range.

In this study, the IoT-Hub network model was designed to support smart farm devices. The IoT-Hub network supports the oneM2M common platform and constitutes the low-level layer of the three-layer FaaS system. The IoT-Hub network was designed under the condition that sufficient power, such as that required by the LoRa Class C platform, is provided [32]. IoT-Hub targets a network in which the device positions are fixed and can be applied to all short-, medium-, and long-distance wireless networks. A user can directly transmit control messages, such as actuator control and data collection commands, to a certain terminal through the server. In addition, the sleep-mode time is synchronized between a given terminal and gateway. Thus, the terminal can receive data from the gateway at a predetermined time without transmission and reception of additional control messages. As a result, the energy efficiency is enhanced. Figure 1 shows a network model to which IoT-Hub can be applied.

IoT devices can either access IoT-Hub through the interface or communicate with the FaaS layer directly. IoT-Hub delivers messages between IoT devices and FaaS, or between IoT devices, based on the transmission control protocol/Internet protocol (TCP/IP). FaaS stores the data collected from IoT-Hub in a database and provides the required functions for the user to implement machine learning algorithms in the application layer. It can also transmit commands to IoT devices through IoT-Hub depending on user requests. Users can monitor the data stored in FaaS via user devices such as computers and smartphones and can transmit messages to IoT devices through FaaS and IoT-Hub.

### 2.2. IoT-Hub Registration Procedure for FaaS

**Figure 2:**
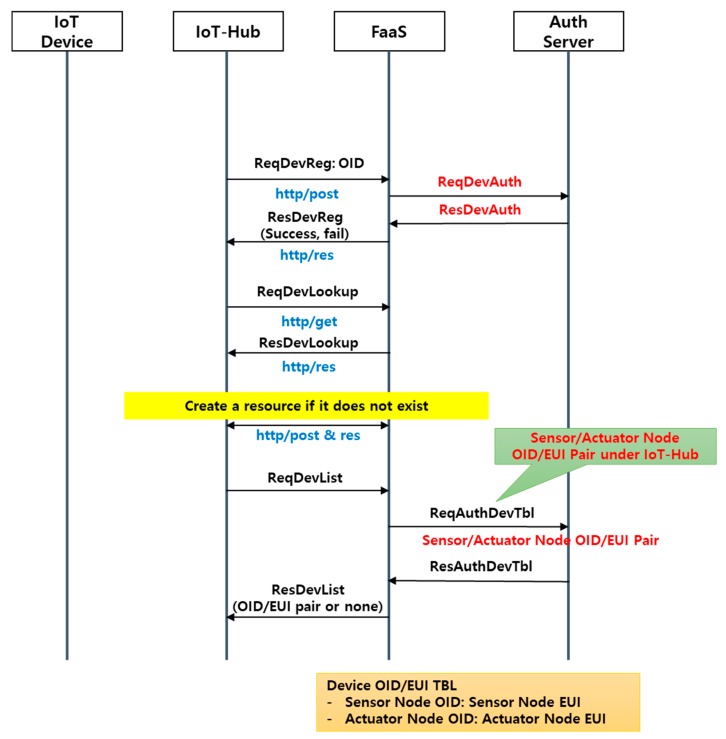
IoT-Hub registration procedure. OID: object identifier; EUI: extended unique identifier.

As is apparent in Figure 2 and Table 1, for IoT-Hub to be linked with FaaS, a registration process that implements the server authentication process using the IoT-Hub object identifier (OID) is required. When the OID authentication step is complete, IoT-Hub requests lookup of a given IoT-Hub resource in FaaS using the OID. If the resource does not exist, the resource is created in FaaS based on the OID. When the IoT-Hub resource creation step is finished, IoT-Hub requests a list of IoT devices connected to FaaS via IoT-Hub (the OID and extended unique identifier (EUI) pair). If IoT-Hub receives the list of IoT devices in a normal manner, the registration process is complete. The registration process is executed whenever power is applied to IoT-Hub, and the resource creation process is omitted if the target IoT-Hub resource already exists in FaaS.

This FaaS, IoT device, and IoT-Hub network process includes processes between each communication entity, such as device registration/deletion, sensing information requests/searches, control information transfer/status checks, and a request for a list of registered equipment, and follows the specifications of the oneM2M platform [33].

## 3. FaaS System

**Figure 3:**
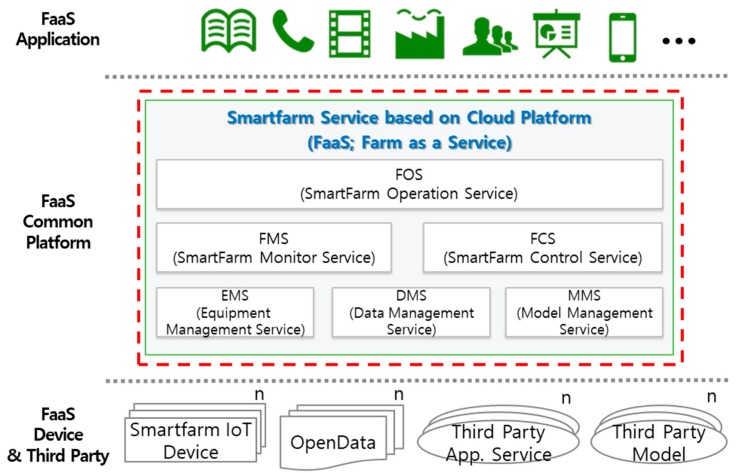
Farm as a Service (FaaS) configuration.

The FaaS system shown in Figure 3 is a PaaS environment in which a smart farm service is provided through virtualization of farm resources horizontally. This system facilitates high-level application services by providing an operation service that supports data collection/control/management and an API service that provides a development environment, as well as farm operation and monitoring, equipment management, data management, and model management [34,35]. FaaS also registers, connects, and manages the devices installed at the farm and provides services for collecting device-sensing and driving information and for controlling these devices. The functions of each component are described in the following subsections.

### 3.1. Equipment Management Service (EMS)

The Equipment Management Service (EMS) supports the installation, modification, removal, and automated connection of the IoT devices, actuators, hybrid controllers, and IoT-Hub installed in a greenhouse, and collects equipment condition and operation information. The detailed functions of the EMS are as follows:

- Registration, modification, removal, and connection of cloud-based equipment installed at the farm;
- Management of driving cycles and errors of cloud-based equipment, such as IoT devices and actuators;
- Checking of firmware versions of equipment, such as IoT devices and actuators;
- Provision of support for automatic/manual upgrade installation of firmware versions of equipment, such as IoT devices and actuators; and
- Storage, maintenance, and management of information for maintenance of corresponding services.

### 3.2. Data Management Service (DMS)

The Data Management Service (DMS) collects the required external data from public data services and records them in a database. The detailed functions of the DMS are as follows:

- Collection of required data from public data services;
- Management of public-data meta information, such as public data name, provider, registration date, and renewal date information;
- Registration, connection, modification, and deletion of collected data from database; and
- Storage, maintenance, and management of information for maintenance of the corresponding service.

### 3.3. Model Management Service (MMS)

The Model Management Service (MMS) applies growth models or crop and facility management models and algorithms developed by the environment control algorithm developers. This service can also make internal FaaS data available for external access. Similarly, external developers can provide third-party application services using the MMS. The detailed functions of the MMS are as follows:

- Provision of the interface through which growth model (or environment control algorithm) developers can register control models in smart farm services;
- Registration and modification of model meta information, such as model name, input value, output value, model execution method (e.g., communication protocol, data format, and operating cycle), and developer information;
- Provision of cloud data user information, such as the user guide, to service developers;
- Setting of authentication and authorization for service developers to access cloud data;
- Collection of usage data, such as the number of calls and MMS transfer capacity by the developer and service; and
- Storage, maintenance, and management of information for maintenance of the corresponding service.

### 3.4. SmartFarm Monitor Service (FMS)

The SmartFarm Monitor Service (FMS) monitors the environmental and driving data of the sensors and actuators collected via the EMS and searches through the stored data. This service continuously measures the farm environment status, so that the results can be collected and analyzed. The detailed functions of the FMS are as follows:

- Monitoring and searching of environmental data inside and outside the farm;
- Collection of environmental data at predetermined times by designating the data collection cycle and zone;
- Provision of notification functions to users and administrators if data are not collected at a predetermined time;
- Provision of status information on equipment installed at the farm; and
- Storage and maintenance of information for maintenance of the corresponding service.
  Features of the growth models include the following:
- A growth model can use data provided by the DMS as input or use separately acquired data;
- A growth model can be operated by acquiring input values in advance using the DMS or by receiving input values during the model execution; and
- The output values of the growth model are stored and managed by the FaaS system.

### 3.5. SmartFarm Control Service (FCS)

The SmartFarm Control Service (FCS) is linked to the EMS and transfers control commands so that actuators can implement user commands. This service can manage the farm in a fully or semi-automated manner if the application of the environment control algorithm is set in the FMS. The detailed functions of the FCS are as follows:

- Transmission of user commands to the EMS and receipt of feedback on the control results;
- Provision of emergency response functions, such as user notifications, in the event of an emergency, such as hardware failure or network disconnection;
- Management of the actuators installed at the farm on a fully or semi-automated basis; and
- Storage and maintenance of information for maintenance of the corresponding service.

### 3.6. SmartFarm Operation Service (FOS)

The SmartFarm Operation Service (FOS) is a comprehensive service that not only records and manages the production management information of the farm, but also collects and displays reporting functions useful for production and management decision making. The FOS can be utilized to computerize the production and management data of the farm and manage the farm and farming activities by collecting and analyzing these data. The detailed functions of the FOS are as follows:

- Provision of a unique household ID, area information, facility house ID, facility house type, and facility house area IDs;
- Manual and automatic input of information on farming work status;
- Provision of farming work status monitoring and lookup service;
- Storage and maintenance of information for comprehensive service maintenance; and
- Provision of an information integration function with separate enterprise resource planning and management information systems for farm operation.

## 4. Implementation of Prediction System

The FaaS disease and pest prediction service can confirm the presence of diseases and pests using disease and pest images captured by capable service terminals. For example, disease and pest management applications and image-capturing devices can be used. This service also provides predictive information on the likelihood of disease and pests, based on operation information obtained from sensors and controllers installed inside and outside the facility buildings. In this study, host plants, pathogens, and environmental factors were analyzed and simulated by implementing data collection, processing, and analysis technologies through FaaS. Hence, it was possible to systematically analyze the complex interrelationships among the components of plant disease occurrence.

Plant diseases cause morphological changes or degrade growth/reproduction functions by changing the metabolic processes and physiological forms of plants. The causes of the various plant diseases include bacteria, fungi, and viruses. Plant diseases occur when certain components are comprehensively quantified; these components can be classified as pathogens, environment-based factors, and host plants. Here, pathogens are modeled by considering the sum of their virulence and density. Similarly, the environment-based factors are quantified as the sum of the conditions (e.g., temperature and humidity) that promote disease occurrence. Finally, the host plants are quantified as the sum of the conditions that foster plant susceptibility.

A disease may occur when the disease onset conditions of each element are established. In contrast, the disease may not occur or the disease onset will be weak if any of the elements are absent. Pathogen virulence and density play an important role in plant disease onset. As for the host plants themselves, the sum of the elements that foster plant susceptibility, which corresponds to the disease onset conditions, is important. In other words, contact between plants and pathogens is required for interaction, and the disease onset conditions must be met after such a contact. If such conditions are changed by an increase or decrease in one of the elements, the disease occurrence degree is dramatically changed [36].

### 4.1. Strawberry Cultivation Test Bed

**Figure 4:**
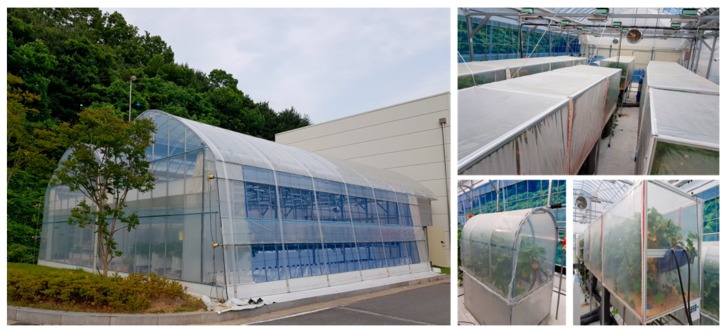
Strawberry cultivation test bed.

In this study, to construct a disease and pest prediction system for Seolhyang, a South Korean strawberry variety, two linked smart greenhouses were constructed, as shown in Figure 4. Inside the greenhouses, 16 chambers with constant temperature and constant humidity functions were designed, and the Seolhyang infection model was established through tests and analysis.

**Figure 5:**
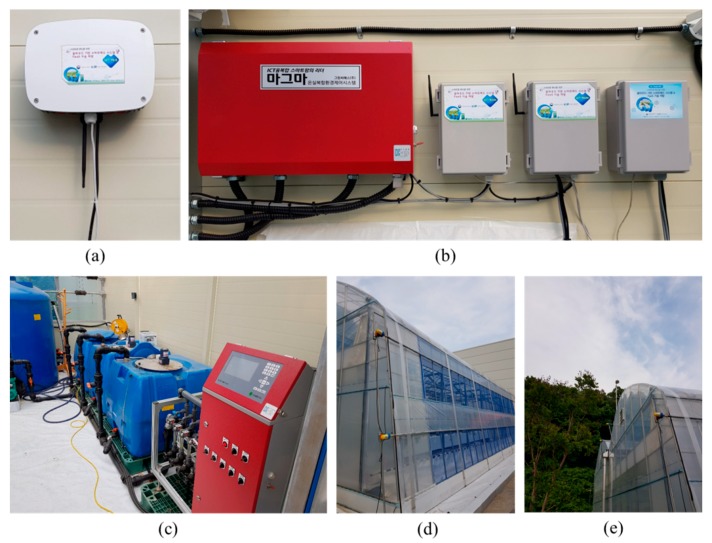
Environmental information collection devices and control devices inside and outside greenhouses: (**a**) IoT-Hub, (**b**) IoT devices, (**c**) nutrient solution supply system, (**d**) side windows, and (**e**) skylight.

In addition, to verify the usefulness of the proposed FaaS system, the FaaS low-level layer equipment was designed by installing oneM2M-based IoT devices as shown in Figure 5. These devices measured the temperature, humidity, CO_2_ concentration, and illumination intensity inside and outside the smart greenhouses. IoT-Hub supporting the LoRa network was also established.

As observed in Table 2, IoT devices based on environmental factors include devices for managing greenhouse environments such as greenhouse main sensors, actuators, or fertilizer controllers. They can be connected with FaaS through IoT-Hub. Wired communication (RS-485, CAN, etc.) is used to communicate with the main sensors, actuators, fertilizer controllers, and the like, while LoRa-based wireless communication is performed with IoT-Hub.

**Figure 6:**
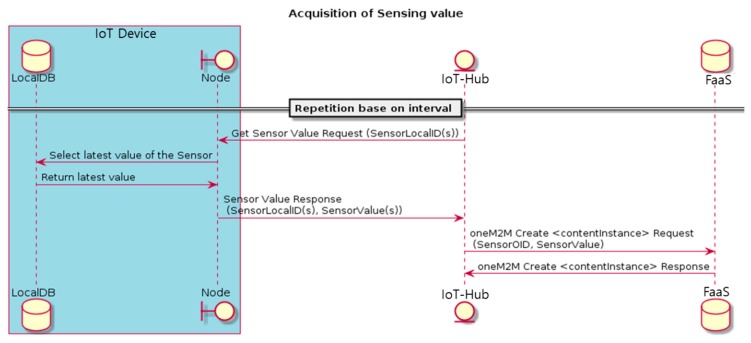
IoT device operation scenario: acquisition of sensing value.

The operation scenario of the IoT device comprises the collection of sensing information and actuator operation. As shown in Figure 6, sensing information is collected at the request of IoT-Hub. IoT-Hub sends sensor value request messages to the IoT device at fixed intervals and selects the target sensor using the Local ID as a parameter. The IoT device receives the request and sends the latest sensor value and sensor Local ID in the sensor value response message. The latest sensor value is an analog value; in the case of a digital sensor, it is converted into an analog value according to a virtual linear rule and transmitted. IoT-Hub receives the sensor value response message and publishes the sensor value to FaaS. FaaS stores the sensor value in the database; in the case of a digital sensor, the transmitted sensor value is first converted back into a digital sensor value and then stored in the database.

**Figure 7:**
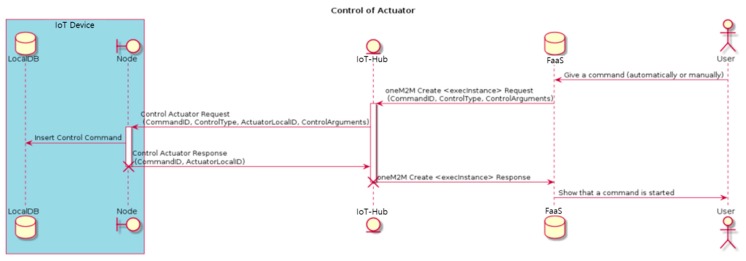
IoT device operation scenario: control of actuators.

As shown in Figure 7, the user can issue control commands for the controller through the FCS of FaaS. The CommandID and control command are published from the FaaS to IoT-Hub. When the latter receives the control command, it sends a control request message, CommandID, ActuatorLocalID, and parameters to be used for control to the corresponding IoT device connected to the controller. The IoT device begins control for the corresponding controller and sends a control response message with the CommandID. Here, the response indicates that the control command was successfully transmitted, not that control was actually started or completed. When IoT-Hub receives the control start message, FaaS publishes the ID of the control command and a message indicating the start of control.

### 4.2. IoT-Hub Communication Performance Evaluation

Unlike other industrial fields, many problems must be overcome by wireless communication in the agricultural field, such as long-distance communication and real-time crop monitoring challenges.

Compared to oneM2M Release 1, Release 2 is focused on various IoT platforms and network connections. Among these, Internet Protocol for Smart Objects (IPSO) is an Internet Protocol (IP)-based protocol of objects that use a small amount of memory and has the advantage of easy expansion between devices and objects [37,38]. We designed a lightweight protocol that provides end-to-end connectivity between IoT devices and between IoT devices and IoT-Hubs, and then, we evaluated the interoperability of oneM2M with IPSO. As IPSO objects have a simple and flexible object model, they can be used in HTTP and other protocols [39,40].

Table 3 presents the stack structure for IoT devices applied in the greenhouse. The existing stack structure provided by the IPSO Alliance was expanded. Each object is separated by a unique ID and has related resources specific to each object. The resources contain information such as object types, values, and units that are also identified by a unique ID.

IPSO-based agricultural IoT systems can be largely divided into IoT devices that collect sensing information and operate actuators, IoT-Hubs that collect information from IoT devices, and FaaS that defines collected information as objects and assigns an IP. The information collected from IoT device sensors is transmitted to IoT-Hub as packets through wireless communication.

IoT-Hub was developed to overcome these problems and facilitate device connection to the cloud. Table 4 gives the technical specifications of IoT-Hub. In this case study, the performance of IoT-Hub was evaluated.

According to the specifications given in Table 5, to test the communication distance, a long-range wide area network (LoRaWAN) module operating at 922.3 MHz (in the industrial, scientific, and medical (ISM) band) was developed and IoT-Hub was constructed. As given in Table 6, the performance of IoT-Hub was tested by measuring the packet delivery ratio according to the distance between IoT-Hub and the test bed in the line-of-sight environment. It was confirmed that stable communication could be achieved at a ratio of 98% or higher on average for distances up to 1.7 km.

### 4.3. Strawberry Disease Prediction Service

The purpose of the FaaS system is to improve crop productivity and provide convenient convergence technology applications for farm households that operate smart farms. In this study, a strawberry disease infection prediction model was developed by implementing the IoT-Hub network layer for wireless communication as well as the FaaS middle layer for data collection, processing, and analysis.

Many models have been developed to predict the infection probabilities of specific strawberry diseases. Among them, the General Infection Model was constructed based on the temperature and wet spell. This model can be used when new and minimally researched pathogens are found [41]. The General Infection Model was developed to explain any disease system when appropriate coefficients are provided. In this model, the critical disease onset limit is defined as 20% disease incidence or a 5% infected leaf area, under the assumption that the number of pathogens is unlimited. The model estimates the wetting duration required to reach the critical disease onset limit at a specific temperature.

As the General Infection Model predicts infection risks according to the weather conditions assuming that there are always sufficient pathogens to cause infection, this model can be applied in various ways to create a disease infection prediction model for strawberries.

In this study, the *Botrytis cinerea* strain was applied to Seolhyang, which is a strawberry variety grown in the largest area of South Korea. The disease occurrence trend according to the temperature and leaf surface wetting duration was then monitored through FaaS [42]. Based on the test results, a model for predicting the *Botrytis cinerea* infection risk of Seolhyang using the General Infection Model was developed.

First, a syringe needle was used to cut the leaves of Seolhyang plants that had been planted in the test bed greenhouse two months earlier. Then, the leaves were sprayed with *Botrytis cinerea* bacterial culture fluid diluted to a concentration of 100 cells/mL. After constant temperature and constant humidity functions were set for the greenhouse chambers, the disease symptoms were examined for three months. Table 7 lists the environmental conditions of each chamber.

The general infection model is expressed as follows:

(1) $$W\left(T\right)=\{\begin{array}{c} \frac{Wmin}{f\left(T\right)},\;\frac{Wmin}{f\left(T\right)}\leq Wmax\\ Wmax,\;\frac{Wmin}{f\left(T\right)}>Wmax \end{array}$$

(2) f(T)={(Tmax−TTmax−Topt)(T−TminTopt−Tmin)(Topt−Tmin)(Tmax−Topt), Tmin≤T≤Tmax    0,    T<Tmin or T>Tmax

Here, W(T) is the wetting duration required to reach the critical disease onset limit at temperature *T* (required wetness duration, time); Wmin and Wmax are the minimum and maximum required wetting durations (time), respectively; f(T) is the infection probability at temperature *T* (0–1); Tmin, Topt, and Tmax are the minimum, optimum, and maximum temperatures for infection (°C), respectively; and T is the average temperature at a given time (°C).

The equation for infection probability f(T) at T (Equation (2)) was originally introduced to explain the effect of *T* on the crop development rate [43]. This model is based on the beta function, which is commonly used as a probability function in statistics to explain a biased distribution. The value of the beta function lies between 0 and 1 because it indicates probability. Therefore, f(T) = 0 holds for *T* = Tmin and *T* = Tmax, and f(T) = 1 holds for *T* = Topt. Figure 6 shows the infection probability and required wetness duration according to the environmental conditions. The infection model for Seolhyang was completed by maintaining the four conditions given in Table 7 within the test bed for three months and analyzing data from the experimental and control groups. 

**Figure 8:**
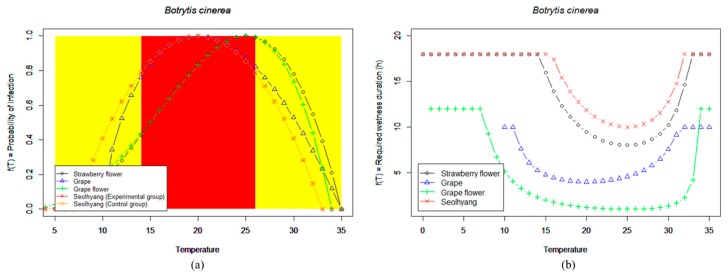
Comparison of general infection models for *Botrytis cinerea*: (**a**) Infection probability according to temperature, and (**b**) required wetness duration according to temperature.

*Botrytis cinerea* of Seolhyang can survive when attached to the surface of plants and when a high concentration of infectious agents, such as bacterial resins that appear on branches in the early stages of growth, exists. Except for hot summers, infection can be easily transferred within or between orchards at any time. Based on the analysis presented in Figure 8a, if the probability of occurrence of greenhouse diseases is determined to be 80%, maintenance of optimal temperatures and wetting durations can be determined using (b). Accordingly, we propose the following regarding disease control:

- Forecast less than 0.8: For agronomic control, (A) adequately manage ventilation; (B) pay attention to watering and avoid excessive humidity; and (C) immediately remove dead, aged, and infected leaves and infected fruit. Note that Seolhyang breeds are more susceptible to gray mold when subject to cold-weather damage compared to other breeds. Extramembranous supplementary heat should be provided.
- Forecast for 0.8–1.0: Treat chemicals registered in strawberries with chemical control according to safety standards. It is also effective to conduct preventive treatment before the disease has occurred. Connect to the pesticide information system to distribute the correct types and amounts of pesticides and conduct disease control [44,45].

In addition, similar curves were obtained for the *Botrytis cinerea* infection model applied to other crops, and it was found that, in comparison with other strawberry varieties, Seolhyang is resilient at low temperatures [46,47].

In the case of the *Botrytis cinerea* disease in Seolhyang, the critical disease onset limit was defined as 20% disease incidence, assuming that an unlimited number of pathogens existed. As for Wmin and Wmax for infection at Topt, the wetting duration that corresponded to 20% disease incidence was calculated after obtaining the relationship between the wetting duration and disease incidence from a regression analysis based on the FaaS MMS data. In addition, Tmin and Tmax were determined considering the disease incidence of Seolhyang *Botrytis cinerea* obtained using the test bed, as well as the results of a density change test.

The reciprocal of W(T), which is the result of the General Infection Model, can be considered as the magnitude of the infection risk increasing at that time, and the accumulated value of the reciprocal of W(T) can be regarded as the magnitude of the possibility that infection succeeds at that time. Therefore, a model that calculates infection risk using the accumulated value of the reciprocal of W(T) was developed. If the leaves are dried continuously, the bacterial activity decreases; thus, the infection possibility disappears. Therefore, the conditions for initializing the infection risk were determined considering changes in the density of the *Botrytis cinerea* on the leaves. 

Analysis of various models through comparison with the control group using the test bed revealed that the models of the crops infected with *Botrytis cinerea* were similar. The disease onset Topt of Seolhyang was approximately 20 °C. The disease incidence decreased very sharply in the temperature range of 15–20 °C and exhibited a relatively slow increase in the temperature range of 20–30 °C. In addition, the disease occurred easily when the conditions within the facility were humid and occurred widely when a sudden drop in nighttime temperature caused cold damage. These results can guide consultations for farm households and technical personnel involved with Seolhyang cultivation using the FaaS disease prediction system, for example, in terms of environmental control parameters for the facility.

## 5. Discussion

IoT is generally perceived as a system in which sensors and data are used to implement advanced processes or activities in industrial or commercial environments. Interestingly, agriculture is a field that can significantly benefit from ICT. This study proposed an FaaS system for a greenhouse, constructed using IoT, cloud computing, big data, and mobile technology. The performance of each layer was evaluated by implementing a disease prediction application. The IoT-Hub system was developed, which supports oneM2M and LoRa, and is optimized for the agricultural environment. This system was improved to reduce unnecessary control messages, namely, to provide communication with minimal messages. In addition, through each function in the FaaS middle layer, a general infection model for Seolhyang, a South Korean strawberry variety, was developed. This example demonstrated that various services, such as device malfunction diagnosis and disease and pest image recognition, can be provided by FaaS, and disease and pest infection models for various crops can be developed.

Advanced ICT convergence technology has made it possible to construct and utilize a system for predicting the occurrence frequency and incidence of pathogens that infect crops. However, to construct prediction systems using vast amounts of climate information and real-time monitoring of rapidly changing climates, it is necessary to develop more advanced prediction models with closer cooperation between communication, network, and software technologies. It is difficult, however, to perfectly understand and predict environmental, host plant, and pathogen patterns, which vary constantly. However, if integrated systems such as FaaS are developed, and various input datasets required for service configuration and interrelationship analyses are collected, more accurate plant disease prediction systems may be constructed using advanced systems.

## 6. Conclusions

FaaS-based smart farms can substantially lower initial facility installation costs and are expected to become a major source of smart farm expansion as they are relatively easy to install through mobile-based services. In the future, smart farm technology development will center around knowledge services that optimize the agricultural industry value chain as well as environmental information collection and facility control. Weather information, distribution information, pricing information, and the like will be collected and analyzed through Big Data and cloud-based systems and provided to agricultural decision makers. Agricultural managers can use this variety of collected and analyzed information to maximize the efficiency of decision making in agricultural production and distribution processes related to crop selection, production volume, disease control and prevention, shipping, and distribution channels. In the future, the developed system will be linked to the National Crop Pest Management System to provide information on infection risk such as risk based on crop, mycelial growth rate, disease development speed, germination rate, and disease outbreak quantity. Further research and development will be conducted for various agricultural technologies, such as technology that can use Big Data pertaining to agricultural environments to predict possible disease outbreak before diseases and pests spread, as well as technology that can use rapid information sharing to distribute pesticides even after an outbreak.

## Figures and Tables

**Table 1:** List of functions for IoT-Hub registration.

Function Name	Factor	Description
ReqDevReg	OID	IoT-Hub registration request
ReqDevAuth	OID	IoT-Hub authentication registration request
ResDevAuth	-	Response to IoT-Hub authentication registration request
ResDevReg	-	Response to IoT-Hub registration request
ReqDevLookup	OID	IoT-Hub lookup request
ResDevLookup	-	Response to IoT-Hub lookup request
ReqDevList	OID	IoT device list request
ReqAuthDevTbl	OID	IoT device authentication list request
ResAuthDevTbl	OID & EUI	Response to IoT Device authentication list request
ResDevList	OID & EUI	Response to IoT Device list request

**Table 2:** Sensor specifications based on environmental factors.

Environmental Factors	EC ^1^	pH	Temperature/Humidity	CO_2_
Measurement Method	AC ^1^ 4-electrode method	pH: 0.0–14.0		Dual Light Wavelength Non-Dispersive Infrared (NDIR)
Measurement Range	0.0–5.0 ms/cm	pH: 0.1 ± 0.1	Temperature: −20–80 °C Humidity: 0–100 %RH ^1^	0–3000 ppm
Measurement Degree	EC ±3% F.S.	Auto 0–100° C	1.00%	±60 ppm, ±3% of measurement
Transmission Output	DC ^1^ 4–20 mA	DC 4–20 mA	-	Normal 65 mA, Peak 170 mA
Power	AC 220 V ± 10% 50/60 Hz	AC 220 V ± 10% 50/60 Hz	-	DC 7–12 V
Dimensions (mm)	L × W × H: 72 × 115 × 90	L × W × H: 72 × 115 × 90	H: 60	L × W × H: 17.6 × 65 × 45

^1^ EC and RH denote electrical conductivity and relative humidity, respectively. DC and AC denote direct current and alternating current, respectively.

**Table 3:** Stack structure of IoT devices applied in the greenhouses.

Ch. ^1^	Item (Unit)	IPSO Object ID	IPSO Object	IPSO Resource ID (Sensor Value)	Sensor Value (Float)	IPSO Resource ID (Unit)	IPSO Resource ID (Application Type)	Application Type
1	CO_2_ (ppm)	3300	IPSO Generic sensor	5700	0–3000	5701	5750	CO_2_
2	Temperature (°C)	3303	IPSO Temperature Sensor	5700	−20–80	5701	5750	Temperature
3	Humidity (%)	3304	IPSO Humidity Sensor	5700	0–100	5701	5750	Humidity
4	Medium Water Content (g)	3322	IPSO Load Sensor	5700	0–100,000	5701	5750	Medium Water Content
5	Nutrient Solution EC (ms/cm)	3300	IPSO Generic sensor	5700	0–10.0	5701	5750	Nutrient Solution EC
6	Waste Nutrient Solution EC (ms/cm)	3300	IPSO Generic sensor	5700	0–10.0	5701	5750	Waste Nutrient Solution EC
7	Feed fluid pH (pH)	3300	IPSO Generic sensor	5700	0–14.0	5701	5750	Feed fluid pH
8	Waste Nutrient Solution pH (pH)	3300	IPSO Generic sensor	5700	0–14.0	5701	5750	Waste Nutrient Solution pH
9	Waste Nutrient Amount (g)	3322	IPSO Load Sensor	5700	0–100,000	5701	5750	Nutrient Solution Amount
10	Waste Nutrient Solution Amount (g)	3322	IPSO Load Sensor	5700	0–100,000	5701	5750	Waste Nutrient Solution Amount
11	Medium Temperature (°C)	3303	IPSO Temperature Sensor	5700	0–60	5701	5750	Medium Temperature
12	Medium EC (ds/m)	3300	IPSO Generic sensor	5700	0–9.99	5701	5750	Medium EC
13	Medium Moisture Content (%)	3304	IPSO Humidity Sensor	5700	0–99	5701	5750	Medium Moisture Content

^1^ “Ch.” denotes channel.

**Table 4:** IoT-Hub specifications.

**Device Specifications**	- AP: Samsung Exynos 4412 ARM Cortex-A9(Q-Core) 1.5 GHz - RF Front-End Transceiver: SX1257 - Concentrator: SX1301 - Frequency: 917–923.5 MHz
**Major Features**	- Listen before talk (LBT) support - Long-term evolution (LTE) model for wireless backhaul (Optional) - Backhaul IPv4 - Global positioning system (GPS) (Optional) - DC5V
**Device Specifications**	- Performance of long-range wide area network (LoRaWAN) concentrator and network server roles - Compliance with LoRaWAN 1.0.2 specifications - Compliance with oneM2M Release 1

**Table 5:** IoT-Hub test environment information.

Category		Packet Delivery Performance Measurement According to Distance
Environment setting	Topology (Test bed: IoT-Hub)	1:1
	Distance	100–MAX m
	Bandwidth	125 kHz
	Code rate	4/5
	Transmit power	14 dBm with 10-dBi antenna
	Spread Factor	7, 9, 12
	Frequency	922.3 MHz
	BW	125 kHz
	Antenna ground height	5 m
	Ack	Turn off
	Retransmission	Turn off
	Panid	0
	Fixed station (transmission) EUI	0 × 000179
	Mobile station (reception) EUI	0 × 000176
Test	Number of packets delivered	1000
	Number of successes	N

**Table 6:** IoT-Hub test results.

Transmitter Information		Receiver Information		Measurements According to Distance							
Location	Spread Factor	Location	Spread Factor								
Fixed station	7	Mobile station	7	Distance (m)	100	500	1000	1700	2000	3000	MAX (3000)
				Ratio	98%	98%	98%	98%	97%	30%	-
	9		9	Distance (m)	100	500	1000	1700	2000	3000	MAX (4200)
				Ratio	98%	98%	98%	98%	98%	98%	30%
	12		12	Distance (m)	100	500	1000	1700	2000	3000	MAX (4200)
				Ratio	98%	98%	98%	98%	98%	98%	60%

**Table 7:** Environmental conditions by chamber for injection target.

Target	High-Level Condition	Low-Level Condition	Chamber Number	Target
*Botrytis cinerea*	Constant (humidity: 40–50%)	Low temperature (less than 18 °C)	1-2-2	Experimental group
			1-2-3	Control group
		High temperature (20–35 °C)	1-2-5	Experimental group
			1-2-6	Control group
	Constant temperature (18–25 °C)	Dry (humidity less than 20%)	1-2-1	Experimental group
			1-3-2	Control group
		Humid (humidity more than 80%)	1-2-4	Experimental group
			1-3-3	Control group
